# Immunogenicity and Cross Protective Ability of the Central VP2 Amino Acids of Infectious Pancreatic Necrosis Virus in Atlantic Salmon (*Salmo salar* L.)

**DOI:** 10.1371/journal.pone.0054263

**Published:** 2013-01-21

**Authors:** Hetron M. Munang'andu, Ane Sandtrø, Stephen Mutoloki, Bjørn E. Brudeseth, Nina Santi, Øystein Evensen

**Affiliations:** 1 Department of Basic Sciences and Aquatic Medicine, Norwegian School of Veterinary Sciences, Oslo, Norway; 2 PHARMAQ AS, Oslo, Norway; 3 AquaGen, Trondheim, Norway; Leiden University Medical Center, The Netherlands

## Abstract

Infectious pancreatic necrosis virus (IPNV) is a member of the family Birnaviridae that has been linked to high mortalities in juvenile salmonids and postsmolt stages of Atlantic salmon (*Salmo salar* L.) after transfer to seawater. IPN vaccines have been available for a long time but their efficacy has been variable. The reason for the varying immune response to these vaccines has not well defined and studies on the importance of using vaccine trains homologous to the virulent field strain has not been conclusive. In this study we prepared one vaccine identical to the virulent Norwegian Sp strain NVI-015 (NCBI: 379740) (T_217_A_221_T_247_ of VP2) and three other vaccine strains developed using the same genomic backbone altered by reverse genetics at three residues yielding variants, T_217_T_221_T_247_, P_217_A_221_A_247_, P_217_T_221_A_247_. These 4 strains, differing in these three positions only, were used as inactivated, oil-adjuvanted vaccines while two strains, T_217_A_221_T_247_ and P_217_T_221_A_247_, were used as live vaccines. The results show that these three residues of the VP2 capsid play a key role for immunogenicity of IPNV vaccines. The virulent strain for inactivated vaccines elicited the highest level of virus neutralization (VN) titers and ELISA antibodies. Interestingly, differences in immunogenicity were not reflected in differences in post challenge survival percentages (PCSP) for oil-adjuvanted, inactivated vaccines but clearly so for live vaccines (TAT and PTA). Further post challenge viral carrier state correlated inversely with VN titers at challenge for inactivated vaccines and prevalence of pathology in target organs inversely correlated with protection for live vaccines. Overall, our findings show that a few residues localized on the VP2-capsid are important for immunogenicity of IPNV vaccines.

## Introduction

Infectious pancreatic necrosis (IPN) is a highly contagious disease causing high mortality in juvenile salmonids and in postsmolt stages of Atlantic salmon (*Salmo salar* L.) after transfer to seawater, and the disease has a worldwide distribution [1]. The causative agent, IPN virus, belongs to the genus Aquabirnavirus in the family *Birnaviridae* and it is a double stranded RNA virus made of segment A which codes for VP2, VP3, VP4 and VP5 while segment B encodes for VP1, the RNA-dependent RNA polymerase [2]. VP2 is the largest protein (60% of genome segment A) [2], [3] and is the major host immunogenic determinant of IPNV [4], [5]. This protein forms the viral capsid which contains conformational epitopes located on surface projections of the hypervariable regions (HVR) recognized by neutralizing antibodies [5]–[10]. Based on its immunogenic properties, the VP2 has been targeted for the development of recombinant vaccines.

Infectious pancreatic necrosis virus causes persistent infections in salmonids [11], [12] and it has been shown that vaccine generated antibodies are not sufficient to clear these persistent infections. Bootland *et al* [13] have shown that inactivated vaccines do not prevent vaccinated fish from becoming carriers of the virus even in the presence of a strong humoral immune response. Similarly, Melby *et al* [12], [14] also reported of a long term co-existence of infecting virus with neutralizing antibodies. Recently, we compared the efficacy of recombinant subunit vaccines encoding VP2, a whole segment A plasmid DNA, PLGA nanoparticle-based vaccine and water-in-oil inactivated whole viral (IWV) vaccines in Atlantic salmon [15] and consistent with other studies [16]–[18], all vaccines failed to eliminate post challenge persistent infections. These results call for studies to identify the critical determinants of host pathogen interaction at the protein level by profiling residues important for induction of neutralizing antibodies. The aim is to understand why IPN vaccines fail. Data obtained by comparing genomic sequences of field isolates have proven useful for identifying residues engaged in host-pathogen interaction and for understanding virulence differences [19]. Characterization of IPNV isolates obtained from different ecosystems has identified differences in amino acids located in the central region of the VP2 capsid. Sequence analysis of field isolates from naturally occurring infections in Norway, Scotland and Chile have shown that IPNV exists as mixed populations of variant strains having amino acid differences on positions 217, 221 and 247 of the VP2 [19]–[23]. The four common variants documented from naturally occurring infections are the T_217_A_221_T_247_, T_217_T_221_T_247_, P_217_A_221_A_247_ and P_217_T_221_A_247_ [19]–[23]. What has not been established is a possible link between virulence and immunogenicity of the different virus strains. Elsewhere [24], avirulent strains have been used as protective vaccines against virulent strains. Although Song et al [25] pointed out that mutation of the T_217_A_221_T_247_ to the P_217_T_221_A_247_ motif attenuates IPNV from a virulent to an avirulent strain, there is no study that documents use of avirulent strains as protective vaccines against virulent strains. Much as coexistence of infecting virus with circulating antibodies has been reported [11], no study pinpoints the immunological basis of IPNV vaccine failure, thus far. In view of these gaps, we used four strains having amino acid substitutions on positions 217, 221 and 247 made by reverse genetics as derivatives of the highly virulent Norwegian Sp strain NVI-015 to make vaccines. We have shown that residues 217, 221 and 247 of VP2 play an important role for the immunogenicity of IPNV vaccines. The virulent strains (TAT) elicited highest virus neutralization (VN) and ELISA antibody titers both for inactivated and live vaccines. Varying antibody responses were not reflected in differences of post challenge survival percentages for the oil-adjuvanted, inactivated vaccines while correlation was found for the live TAT and PTA vaccines. We found that virus carrier titres were higher in vaccinated fish with lower VN titres. Similarly, the prevalence of pathology in target organs was greater in those fish vaccinated with the least protective vaccines. Thus, a few residues on the VP2-capsid are important for immunogenicity of IPNV vaccines.

## Materials and Methods

### Cells and Viruses

Viruses used for vaccine production, challenge, ELISA antigens and virus neutralization test (VNT) were all propagated on rainbow trout (*Oncorhynchus mykiss*) gonad cells (RTG-2, ATCC CCL-55) at 15°C in Leibowitz L-15 media supplemented with 10% fetal bovine serum (FBS)(Sigma), 2% L-glutamine (Sigma) and gentamycin (Sigma) 25 μg ml^−1^. All viruses used in this study were produced by reverse genetics as derivatives of the highly virulent Norwegian IPNV Sp strain NVI-015 (GenBank accession nos. AY379740) using the method described below.

### Construction of Recombinant Viruses

Generation of full-length cDNA clones of the entire coding and non-coding regions of NVI-015 RNA segment A and B was performed according to procedures described by Yao and Vakharia [26]. The construction of recombinant viruses rNVI-15TAT and rNVI-15PAA has been described elsewhere [27]. Briefly, combining transcripts of pUC19NVI15A plus pU19NVI115B resulted in the recovery of the viral progeny designated as rNVI-15TAT while combining pUC19NVI15VP2 plus pUC19NVI15B resulted in recovery of progeny rNVI-15PAA. Progeny rNVI-15TAT has similar residues (T_217_A_221_T_247_) to the parent IPNV strain NVI-015 while progeny rNVI-15PAA [25] was substituted by T217P and T247A giving a PAA VP2 motif. Once the genetically engineered viruses were made, they were propagated on RTG-2 monolayers on which they have been shown to replicate equally [19] and supernatants were harvested following at 2500 rpm centrifugation and sterile filtration using 0.22μm filters (Whatman GmbH). RNA extraction using the QIAamp viral mini kit (Qiagen) was carried out using the manufacturer's recommendation. Complete nucleotide sequences of segment A and B of each virus was determined as before [19]. Chromatograms were analyzed to ensure that the generated clones had the correct single peaks at positions 217, 221 and 247.

### Plaque purification Assay

To generate additional viruses with a mutation in residue 221 of VP2, we took advantage of previously observed cell culture adaptation attenuation characteristics of the rNVI-15TAT and rNVI-15PAA strains where attenuation is seen in CHSE-214 but not in RTG-2 cells after 10 passages in culture [19]. Parent strains rNVI-15TA and rNV-15PA were passaged 10 times on CHSE-214 cells to yield progeny viruses designated rNV1-15TTT and rNVI-15PTA representing amino acid substitution of A221T. After the 10th passage, both isolates were plaque purified by inoculating RTG-2 monolayers grown on six well plates at 10-fold dilution (10^−3^ to 10^−8^) of cell culture supernatants and adsorbed for 1hr at room temperature (RT). After adsorption the inoculum was removed and cells were overlaid with 0.8% SeaPlaque Agarose (BioWhittaker) in L-15 medium containing 5% FBS and 1% L-glutamine. Cells were incubated at 15°C for 4 days and plaques formed by cytopathic effect (CPE) were removed from the wells using punch biopsy equipment. Plaques were inoculated in RTG-2 monolayers and incubated for 7 days at 15°C. When full CPE was observed, the supernatant was harvested after 2500 rpm centrifugation and sterile filtration as described above. RNA was extracted using the QIAamp Viral RNA mini kit following the manufacturer's recommendations. Complete nucleotide sequences of segment A and B of each virus were determined as described before [19], [25],[28]. Chromatographs were checked to ensure a “clean” ATT codon encoding Thr_221_ in the virus isolate used while variant genotypes used in the study are shown in Table 1.

**Table 1:** Amino acid combinations of the VP2 capsid protein of the different virus strains used to generate the different inactivated vaccines used in the study.

Virus strains	VP2 amino acid residues		
	217	221	247
rNVI-15TA^a^	T	A	T
rNVI-15TT^b^	T	T	T
rNVI-15PA	P	A	A
rNVI-15PT	P	T	A

a The virus strains have a first letter r- to indicate recombinant strains were generated by reverse genetics. ^b^Vaccine strain rNVI-15TT was generated by serial passage of rNVI-15TA and rNVI-15PT was similarly generated through passage of rNVI-15PA, both on CHSE-214 cells (see Materials and Methods for details).

### Preparation of Vaccines

Inactivated vaccines were made by inoculating the different recombinant viruses TAT, TTT, PAA and PTA onto RTG-2 cells to ensure that no mutations occurred during virus culture [25] and were only harvested when total CPE was observed. After centrifugation and sterile filtration, supernatants were inactivated using 0.5% formalin for a minimum of 48 hours at RT and concentrated 8–10x using a standard filtration method at 100kD cut-off. All vaccines were constituted to an equal concentration of VP2 proteins using a standard ELISA procedure [14], equivalent to 1.0×10^9^ Tissue Culture Infectious Doses_50_/ml (TCID_50_/ml) of vaccine. In this ELISA we used the monoclonal antibody AS-1 [29], [30] whose binding ability includes the conserved region of the VP2 capsid outside hypervariable regions (HVR) encoding mutations introduced by reverse genetics in this study (Table 1). In this way all strains used in the present study were constituted at the same concentration because they had the same binding ability in the conserved region of the VP2. Inactivated vaccines were prepared as oil-based emulsions (water-in-oil) and the adjuvant used was mineral oil. Emulsification of the antigens with adjuvant was done using a homogenizer with a standard emulsification stator/rotor connected to an emulsor screen. The oil-based antigen preparation was formulated as water-in-oil (w/o), where the water phase (containing viral antigens) was dispersed into an oil phase (continuous phase containing emulsifiers and stabilizers). Sterility, safety tests, potency and quality assurance procedures were all done according to specifications outlined by PHARMAQ AS, Oslo.

Two viral strains encoding the TAT and PTA motifs produced as described above were used to prepare two live vaccines.

### Experimental Designs

To ensure scientific validity and consistency of our findings, we carried out two independent vaccine efficacy trials using an inactivated and a live vaccine approach. In study I we used four oil-based inactivated vaccines based on viral strains in Table 1 while in study II we used two live vaccines made from the most distant strains encoding the virulent and avirulent motifs (Table 1). In each trial, we used a three parallel tank system using the same challenge virus, injected at the same concentration and proportion of virus shedders (12%) of the total biomass per tank in a cohabitation model. Details of the setup for each efficacy trial are shown below.

Study I was carried out at VESO, Namsos, Norway using inactivated vaccinated made from the four strains shown in Table 1. Atlantic salmon parr, AquaGen AS breed, IPN susceptible strain hatched and reared at VESO's hatchery were used. Fish were divided into five groups of which four were for the inactivated vaccines groups (Table 1) while the fifth group was for unvaccinated control fish. Each vaccine was allocated a total of 186 fish and each fish was injected intraperitoneally with 0.1 ml of the vaccine. After vaccination, 62 fish were transferred to each of the three parallel tanks (Fig. S1). Another 186 control fish were injected with phosphate buffered saline (PBS) of which 62 were transferred to each of the three parallel tanks. The total number of fish per tank was 310 comprising of 62 fish per vaccine by four vaccines plus the PBS injected controls. Study fish were kept in tanks supplied with UV-treated water and fed commercial diets *ad libitum* (EWOS Micro, Bergen, Norway). After vaccination, fish were kept at 12°C (12 hrs darkness and 12 hrs light) for smoltification (preparation for transfer to seawater). Post vaccination samples were collected at 10 weeks post vaccination (wpv; Fig. S1). At each time point 18 fish per group were sampled by removing six fish representative of each group from the three parallel tanks which reduced the total biomass to 250 fish per tank after the second post vaccination sampling.

After 840 degree days of immune induction (10 weeks), fish were transferred to sea water and challenged using a cohabitation model by adding 30 virus shedders per tank (Fig. S1). Virus shedders were injected intraperitoneally with 0.1 ml/fish of strain TAT equivalent to 1×10^7^TCID_50_/fish and were immediately placed in the tanks to cohabit with vaccinated and control fish. One additional parallel tank (not shown in Fig. S1) was injected with one log_10_ lower of the challenge dose. Post challenge samples were collected at four and 10 weeks post challenge (wpc) and 12 fish were sampled per group. Fish were anaesthetized using methiomidate during immunization and sampling.

In study II, two live vaccines were used based on the virulent TAT and avirulent PTA strains. Efficacy trials for live vaccines were carried out at the Aquaculture Research Station of the University of Tromsø, Norway using a standard IPN-susceptible AquaGen breed of Atlantic salmon parr. Virulent strains can be used for immunization at parr stage since post-exposure mortality will not occur at this physiological stage. For identification, each fish was intraperitoneally implanted with a pit-tag number using a pit-tag applicator. Each tag number was electronically registered in an Excel sheet using an automated electronic reader (AEG ID, ARE H5-ISO, Germany) linked to a computer. Fish were divided into three groups, the two vaccines and unvaccinated controls (Fig. S2). Each vaccine group was allocated 138 fish. After vaccinating with live virus vaccine administered at 0.1ml/fish at a concentration of 1×10^5^TCID_50_/fish, 46 fish were transferred to each of the three parallel tanks. Another 138 fish were injected with PBS as controls and were distributed equally into parallel tanks (Fig. S2). To avoid cross infection between fish immunized with different strains of the live vaccines during the study period, each vaccine group was assigned its own three parallel tank system. After vaccination, fish were subjected to the smoltification for 540 degree days. They were fed *ad libitum* commercial feed (Skretting AS, Norway) using automated equipment.

At 8 wpv, fish were challenged using a cohabitation model by adding eight virus shedders in each tank. Virus shedders were injected with 0.1 ml/fish of strain TAT at a concentration of 1.0×10^7.0^ TCID_50_/fish and were immediately put to cohabit with vaccinated fish in the tanks. In addition to virus shedders, each tank of the TAT and PTA vaccinated groups was allocated 30 unvaccinated control fish at challenge (Fig. S2). Control fish could only be added at the time of challenge to prevent their exposure to vaccine viruses during immune induction. When fish stopped dying at the end of the challenge period, survivors of each parallel group were put in one one tank (Fig. S2). Samples were collected at 4 and 8 wpv during immune induction period while post challenge samples were collected at 8 and 17 wpc during the persistent infection period. Samples were also collected during the challenge period (Fig. S2).

In both the inactivated (study-I) and live (study-II) vaccine efficacy trials, samples collected included whole blood in EDTA while head kidney, liver, spleen and exocrine pancreas samples were stored in 10% formalin. Head kidney samples were also stored in transport media for virus cultivation.

### Virus re-isolation and Sequencing

Head kidney tissues were homogenized in a stomacher in transport media followed by a low spin at 2500 rpm for 10 min. 0.1 ml was inoculated on RTG-2 cells grown in 24 well plates to final dilutions of 1 and 0.1%. The plates were incubated at 15°C. After seven days, 0.1 ml of the supernatant was obtained and used to inoculate new monolayers of confluent cells for a second passage. Final reading was carried out after seven days of the second passage. Results were scored based on the presence (positive) or absence (negative) of CPE during the second passage. Supernatants obtained from second passage monolayers were tested for the presence of IPNV using RT-PCR with primers A-Sp500F (5′-GAGTCACAGTCCTGAATC-3′) and A-Sp1689R (5′-AGCCTGTTCTTGAGGGCTC-3′). RNA extraction was carried out using the RNAeasy Mini kit (Qiagen) according to manufacturer's recommendation. PCR products were sequenced as described by Santi *et al* [28] using primers A-Sp500F and A-Sp1689R.

### Virus Neutralization test (VNT)

Sera from experimental fish were diluted in a two-fold dilutions starting from 1∶10 up to 1∶1280 in L-15 medium without FBS and were mixed with an equal volume of virus suspension (10^2^ TCID_50_/ml) in titration plates. The virus-antisera mixture was incubated at RT for 1h. Growth media was removed from RTG-2 monolayers in 96 well plates and replaced with 0.1 ml of the virus-antisera mixture. All serum samples from the four vaccine and control groups were tested against the four viral strains to yield a cross neutralization assay. Positive IPN virus controls, negative controls (plasma and blanks with L-15 medium) were used on each plate. The plates were incubated at 15°C and were examined for presence of CPE after seven days. Replication patterns and kinetics of the virus strains have been characterized previously and it has been shown that their ability to replicate in vitro does not differ between the recombinant viruses [19].

### Enzyme Linked Immunosorbent Assay (ELISA)

ELISA was carried out using the protocol described before [15]. All viral antigens were used at a concentration of 1.0×10^5^TCID_50_/ml while test sera were used at 1∶50 dilution in PBS.

### Histopathology and Immunohistochemistry

Head kidney, liver, spleen and pancreas samples preserved in 10% phosphate buffered formalin were used for histopathology and immunohistochemistry (IHC) examination as previously described [15], [31], [32].

### Statistical analysis

Experimental data was entered in Microsoft Excel^TM^ and later transferred to STATA version 12 (http://www.stata.com) for analysis. The Kaplan Meyer's survival analysis was used to generate the post challenge survival percentages (PSCP) [33]. The cox proportional hazard model was used to determine hazard risk ratios while post challenge relative risk of infection was used to estimate the risk of post challenge virus carrier state for the different vaccines after challenge. Analysis of variance (ANOVA) was used to compare significant differences in the serological titers from VNT and ELISA obtained at various time points. All tests were considered significant for p<0.05 at 95% confidence interval.

## Results

### All inactivated vaccine strains induce high levels of protection against mortality

**Figure 1:**
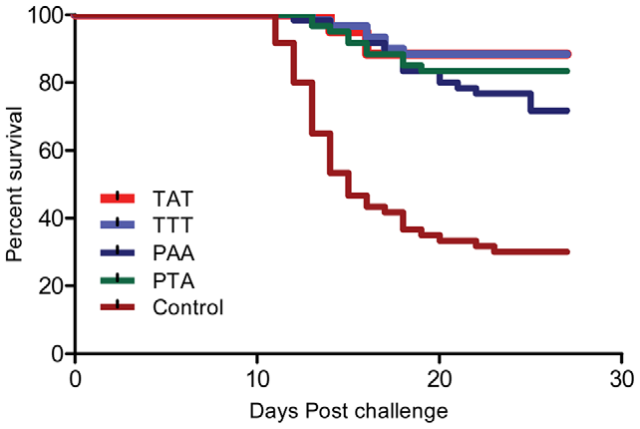
Post challenge survival plots for inactivated vaccines. Kaplan Meyer's (KM) post challenge survival percentages (PCSPs) of fish vaccinated with inactivated, water-in-oil adjuvanted vaccines made from the TAT, TTT, PAA and PTA strains, challenged at 10 weeks post vaccination with the virulent TAT strain. Mortality started on day 11 post challenge and stopped on day 27 when fish stopped dying and the PCSP curves plateaued. The challenge study was stopped on day 31. TAT and TTT groups overlap while there was a reduced survival in the PTA and PAA groups with significantly lower PCSP for the latter (p = 0.042).

Fish vaccinated with the TAT and TTT vaccines had the same level of protection while those vaccinated with the PTA strain had slightly higher mortality (Fig. 1, Table S1). However, hazard risk (HR) ratios did not show statistical differences between the TAT, TTT and PTA vaccinated groups (Table S1). Conversely, fish vaccinated with the PAA vaccine were 2.5 times (HR = 2.4906, 95%CI 1.0328 – 6.0063, p<0.042) at risk of dying than fish vaccinated with the TAT/TTT vaccines (Table S1). Mortality was first observed within three days after challenge among the virus shedders while among the vaccinated and control groups it was delayed until 11 dpc (Fig. 1). Duration of mortality was shortest in the TAT followed by the TTT vaccinated group and it lasted 17 days for the entire study (Fig. 1). The risk of fish dying in the control group was 10 times higher (HR = 10.12, 95%CI 4.5350 – 22.5890, p<0.000) than the TAT vaccinated group (Table S1).

### TAT is the most immunogenic vaccine strain

**Figure 2:**
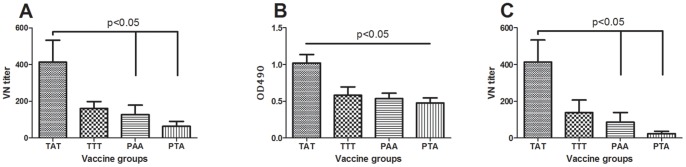
Virus neutralization titers and ELISA antibodies for inactivated vaccines. **A**) Virus neutralization (VN) titers generated from TAT, TTT, PAA and PTA inactivated vaccines against the TAT challenge strain at 10 wpv shows significantly higher VN titer for TAT over PAA and PTA groups (p<0.05). The TTT group was not different from any other group. N = 12; ±SEM. **B**) Antibody response by ELISA at 10 wpv against TAT antigen coat for the TAT, TTT, PAA and PTA vaccines. TAT is different from all other groups. N = 12; ±SEM. **C**) VN titers of serum from fish vaccinated with different vaccines at 10 wpv. Each group of vaccine was tested against the corresponding vaccine antigen. As can be seen the trend is similar to what is seen in B) but with lower antibody levels against corresponding antigen. N = 12; ±SEM.

Based on the observation from the challenge study we were first interested in assessing the antibody levels to the challenge virus (TAT) for the 4 different vaccine groups and we examined the neutralizing titers at 10 wpv (time of challenge). VN titers elicited by the TAT-vaccine (against TAT strain) were significantly higher than the PAA and PTA vaccinated fish (Fig. 2A). The TTT strain was not different from any of the other groups (Fig. 2A). The most distant PTA group (mean titer = 63.33, SEM = 27.03) had six times (6.56) lower VN titer than the TAT-vaccinated fish (mean titer = 413.33, SEM = 120.14) but despite this, no significant difference in survival proportions was observed (Fig. 1). No antibody responses were detected in sera obtained from control fish (not shown).

 We also examined antibody responses generated by the four vaccines against the challenge strain (TAT) by ELISA. The findings corresponded with VN assay results (Fig. 2B) showing that TAT vaccinated fish had a significantly higher antibody level compared to fish vaccinated with the other vaccines at challenge (Fig. 2B). There was no difference between the TTT, PAA and PTA groups. Put together, VN and ELISA data (Figs. 2A, B) with survival data (Fig. 1) show that antibody levels were not predictive of the outcome of PCSPs, apart from the PAA group that was marginally less well protected and also had lower antibody levels than the TAT vaccinated fish (Table S1). Finally, we examined the VN antibody levels against the corresponding virus for all vaccines and the trend observed was not different from what was found when testing against the challenge virus (Fig. 2C), VN titers levels were lower (not significant) for the TTT, PAA and PTA vaccines. Indications are that the TAT strain is the most immunogenic of those tested under the conditions used.

### Higher circulating antibody levels lead to reduced prevalence of virus post-challenge

As our next approach we determined infection rates in head kidney post challenge in surviving fish. We were interested in understanding whether antibody levels at challenge could be used as predictors of the outcome of post challenge infection. Table S2 shows that fish vaccinated with the TAT vaccine (homologous to the challenge virus) had less virus positive head kidney samples at 4 and 10 wpc compared to fish vaccinated with heterologous vaccines. Correlating antibody levels at challenge (Figs. 2A, B) with post challenge head kidney infections (Table S2) show a highly significant negative correlation (r^2^ =  −0.959, p<0.021) at 10 wpc indicating that there was an inverse relationship between antibody levels and post challenge infection. Supplementary data from a low challenge dose (1×10^6^TCID_50/_ml) trial showed absence of post challenge head kidney infections in fish vaccinated with the TAT vaccine while fish vaccinated with heterologous vaccines showed increasing infection rates that corresponded with increase in amino acid substitution on the vaccine strains ( Table S3). Further to this, the risk of post challenge infections was five times (4.8156) higher in fish vaccinated with heterologous PAA and PTA vaccines (RR = 1.2564, 95%CI 1.0396–1.5184, p<0.0182) than fish vaccinated with the homologous TAT vaccine (RR = 0.2609, 95%CI 0.0977–0.6963, p<0.0073) to the challenge virus at 4 wpc (Table S2). The TTT group was intermediate (RR = 1.0256; Table S2). Overall, these data demonstrate that antibodies generated by homologous vaccines to the challenge virus confer the highest protection against infection while those from heterologous vaccines allow for high post challenge infection rates in head kidney but although infection levels are not indicative of protection against mortality.

### Higher antibody levels result in lower infection rates post-challenge

**Figure 3:**
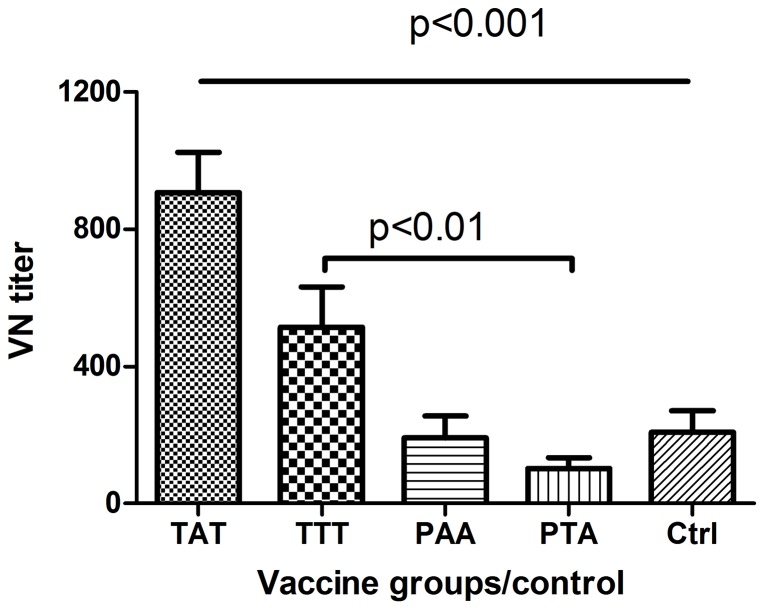
Virus neutralization titers post challenge. **A**) VN titers at 4 weeks post challenge (wpc) show that the TAT vaccinated fish are significantly higher than all other vaccine groups while the TTT group is higher than the PTA fish. Surviving control fish (Ctrl) also show VN titers at 4 wpc and is not significantly different from the TTT, PAA and PTA groups. There is significant increase in the TAT and TTT groups. N = 12; ±SEM. * = p<0.05.

Since antibody levels were found to correlate inversely with virus positivity post challenge we became interested in the kinetics of the immune response post challenge. We found that the TAT vaccinated fish had a significantly higher VN titer than all other vaccine groups at 4 wpc (Fig. 3). At 10 wpc there was no significant difference for VN titers for all groups except the TTT vaccinated fish that had lower titers (Fig. S3). These findings show that post-challenge responses were more rapid in the TAT vaccinated fish, homologous to the challenge virus, than in fish vaccinated with virus strains heterologous to the challenge strain, resembling a boost response in higher vertebrates. These results suggest that the increase in VN titer in all groups was induced by the challenge virus, despite the fact that few fish were found virus positive in the head kidney at 4 wpc. In the TAT vaccinated fish, the level of virus replication in these fish was sufficient to raise the antibody levels after challenge. For the other vaccine groups that were found with high virus prevalence the response was much slower, more like a primary response.

### The live TAT vaccine confers superior protection over a PTA-based vaccine

**Figure 4:**
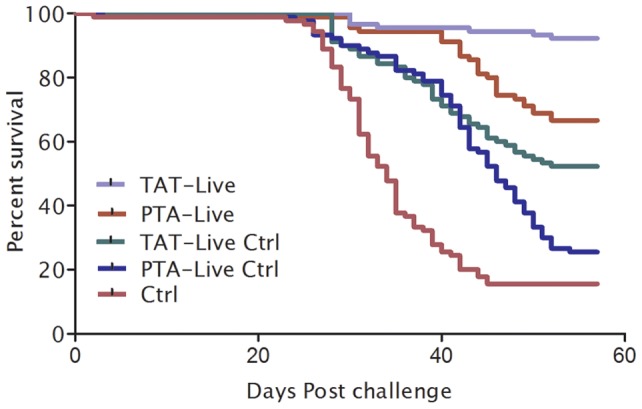
Post challenge survival plots for live vaccines. Kaplan Meyer's (KM) post challenge survival percentages (PCSPs) of fish vaccinated with the TAT- and PTA-live vaccines. TAT-live Ctrl and PTA-live Ctrl are non-vaccinated controls cohabiting with the respective vaccine groups, while the mortality controls (Ctrl) are the reference mortalities in the tanks not including vaccinated fish (cf. Fig. S2). Mortality started on day 21 post challenge and lasted until day 55, and the experiment was stopped 57 days post challenge. PCSP for the PTA-live was significantly lower than for the TAT-live group (p<0.0001).

We included two live vaccine groups in this study, one homologous vaccine to the virulent challenge strain (TAT) and the most distant strain in terms of amino acid substitutions, the PTA variant. Mortality in the virus shedders started 3 dpc while in the controls and vaccinated groups it started 21 dpc and ceased at 55 dpc (Fig. 4). Fish vaccinated with the TAT vaccine had significantly higher protection than the PTA group. Table S4 shows that the mortality risk of fish vaccinated with the PTA vaccine was five times (HR = 4.745, 95%CI 2.084–10.805, p<0.000) higher than fish vaccinated with the TAT vaccine.

### The live vaccines establish viral persistence post vaccination

Since differences in protection obtained for the two vaccine viruses can be influenced by the infection established by the vaccine viruses, we monitored the persistence and viability of the live vaccines up to 8 wpv. Both live vaccines persisted as viable virus at a rate of 100% (*n* = 10) in immunized fish during immune induction up to 8 wpv (Table S5).

### Level of antibodies correlate with protection for live IPN vaccines

**Figure 5:**
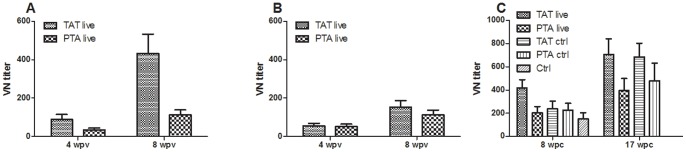
Virus neutralization titers in live vaccine groups. **A**) VN titers against TAT strain for the TAT- and PTA-live vaccinated fish increased markedly from 4 to 8 weeks post vaccination (wpv). **B**) VN titer against the PTA strain was also examined and showed that the homologous titer for the PTA vaccinated fish were equal to their cross-reactivity to the TAT variant. **C**) VN titers at 8 and 17 weeks post challenge (wpc) were examined and while the TAT-live show no increase from time of challenge (A) there is an increase for the PTA-live group. Control fish cohabiting with the vaccinated fish also show an increase. By 17 wpc there is a marked increase in all groups. N = 12; ±SEM.

Neutralizing antibodies of TAT vaccinated fish were higher than for the PTA group prior to challenge when tested against the challenge strain (Fig. 5A). Antibody levels increased during immune induction from 4 to 8 wpv for both the live TAT and PTA vaccinated groups, most pronounced for the TAT group when tested against the TAT-virus (Fig. 5A). When the PTA-live vaccinated group was tested for neutralization against the homologous PTA vius (Fig. 5B), the VN titer was equal to that in the anti-TAT group (Fig. 5A). These findings are similar to what was seen for the inactivated vaccines (Figs. 2B and C) which indicate that the PTA strain was less antigenic than the TAT strain and correlate well with the findings for the inactivated vaccine preparations We also examined the post challenge VN antibodies to assess the “boost” effect of the challenge virus. By 8 wpc, VN titers in the TAT vaccinated fish had not risen above levels detected at challenge while the PTA group showed an increase (Fig. 5C). Despite so, antibody levels of the PTA vaccinated fish at 8 or 17 wpc did not rise to levels equal to the TAT vaccinated fish (Fig. 5C). These findings correspond with what was seen for the inactivated vaccines. We also determined the level of antibodies generated from fish vaccinated with the live TAT and PTA vaccines using ELISA and there was a significant difference (p<0.0001) between the two groups (Fig. S4). Both groups showed a similar trend of increasing antibody levels as for the VN titers and the TAT vaccinated fish had higher antibody levels than the PTA group when tested against the TAT antigen during immune induction (Fig. S4). At challenge (8 wpv), antibody levels from the TAT vaccinated fish (OD_490_, mean titer = 1.764, SE = 0.293) were five times (4.927) higher than levels detected in the PTA vaccinated fish (OD_490_ mean titer  = 0.358, SE = 0.213) when tested against the TAT antigen. These findings show that antibody levels detected at challenge were predictive of the risk of post challenge mortality. In addition, the risk of PTA-controls (HR = 13.8165, 95%CI 6.334-30.1361, p<0.000) dying was about five times higher than TAT-controls (HR = 8.1428, 95%CI 3.6616–18.1079, p<0.000) suggesting that there was a protective effect from vaccinated fish on controls, likely a herd immunity effect. The risk of fish dying in the control group that did not cohabit with vaccinated fish was 29 (HR = 29.3723, 95%CI 13.4755–64.0222, p<0.000) times higher than the TAT vaccinated fish. In summary, these data demonstrate that the risk of post challenge mortality for live vaccines can be predicted by antibody levels detected at challenge using a homologous antigen to the challenge virus.

### Live TAT vaccines protect against pathology in target organs (pancreas and liver)

**Figure 6:**
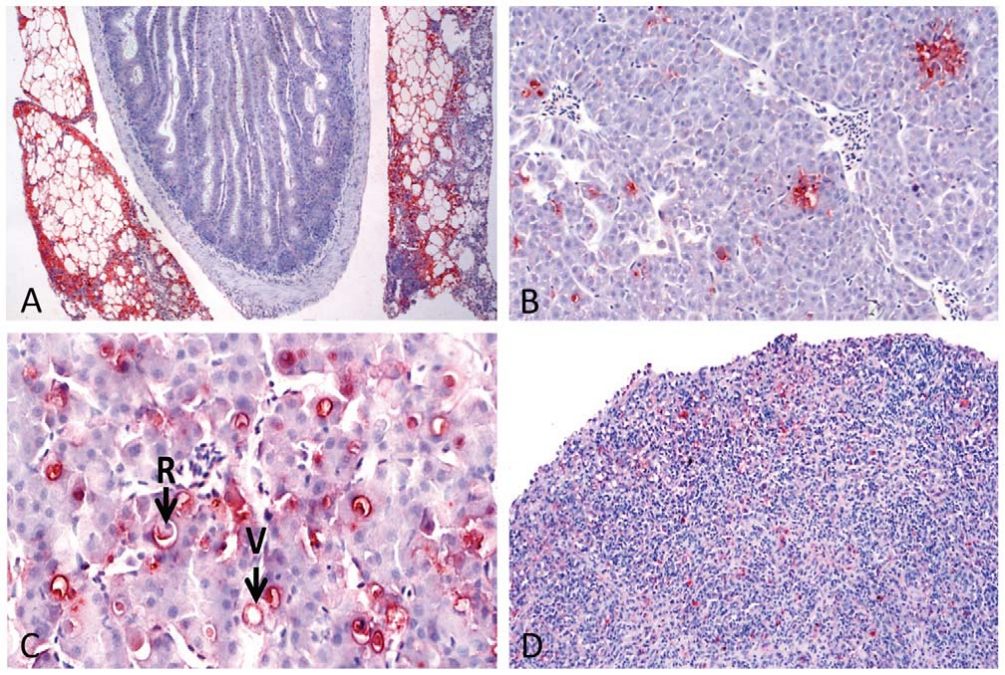
Immunohistochemistry (IHC) of infected tissues. Detection of viral antigens in infected tissues by IHC is depicted as red stain. **A)** shows extensive distribution of viral antigens in exocrine pancreas. **B)** Viral antigens in liver parenchyma of multifocal appearance. **C)** Rounding (R) of individual liver cells and also with vacuolation (V) concomitant with positive staining for virus (red). **D)** Viral positive cells scattered in spleen tissue.

In order to gain insight on mechanisms of protection rendered by live vaccines we assessed their ability to prevent establishment of pathology in target organs. Viral antigens were only detected in infected tissues after challenge and not before (Table S6), showing that the live vaccines did not replicate to levels giving pathology in internal organs or detectable by IHC. Further, this implies that viral antigens detected after challenge were due to replication of the challenge virus. In the pancreas and liver, distribution of viral antigens was diffuse covering most of the parenchyma (Fig. 6A, B). In the liver it formed necrotic foci (Fig. 6B) and necrosis of individual hepatocytes with rounding of cells and vacuolation (Fig. 6C). In the spleen and head kidney viral antigens were localized to endothelial lining of vessels and individual cells (likely macrophages) of the interstitium (Fig. 6D). Table 6 shows that no viral antigens and tissue damage was detected from TAT vaccinated fish after challenge while 40% (*n* = 10) of the PTA vaccinated fish had tissue damage in the liver and pancreas. Put together, data in Table S6 and Figs. 3 and 5 show that antibody levels detected against the challenge TAT virus using ELISA and VN predict the outcome of tissue damage in vaccinated fish. In conclusion, we have shown that fish vaccinated with the TAT-live vaccine were protected against tissue damage and that protection correlates with antibody levels detected before challenge when tested against the challenge-virus.

### Confirmation of virus pathogen by sequence analysis

In order to demonstrate that the mortality observed was caused by the challenge virus (strain rNVI-15TA) we extracted RNA and prepared cDNA from supernatants obtained from virus re-isolation studies. Chromatograms from cDNA sequences showed clear peaks encoding T_217_A_221_T_247_ (not shown). Similarly, samples from virus re-isolation at 4 and 10 wpc had the same residues at the defined positions clearly indicating that there was no mutation observed during mortality or in the virus carriers after challenge. Both virus re-isolation and sequencing gave the same results with only one sample of the post challenge studies detected positive on RT-PCR failed to show CPE after the second passage on RTG-2 monolayers (*n* = 180).

## Discussion

One key finding of this study was that IPN vaccines based on the virulent TAT virus variant elicited the highest antibody responses compared to other, lesser virulent virus strains. This was clearly shown from neutralization antibody tests and ELISA for both inactivated and live vaccines. Our conclusion is that the TAT strain is the most immunogenic of those tested while the PTA strain was the least immunogenic. Yet, fish immunized with different variants of IPNV with adjuvanted, inactivated vaccines were equally protected against lethal challenge, although marginally lower for the PAA variant. Using live vaccines, VN antibody titers correlated with protection against mortality. Although less immunogenic, antibodies elicited by PTA vaccines from both the inactivated and live vaccines were neutralizing against the challenge strain. Epitope mapping for IPNV has been reported [5], [7], [9], [29], [30] and these studies point to the VP2 capsid as the immunogenic protein, but these studies do not localize the immunogenic domain. Our findings suggest that there are cross neutralizing epitopes shared by the two most distant strains outside those governed by residues 217, 221 and 247. Nevertheless, the results reported here confirm and provide new insights regarding the role of a few residues in the central region of the VP2 capsid in determining the immunogenicity of IPNV Sp strains.

It has been pointed out [34] that in many cases, protective antigens are not known, and under such circumstances selecting the most promising vaccine candidate based on immunogenicity is an option. In the present study we linked immunogenicity studies and antibody responses with PCSPs as a way of identifying the most protective vaccine variant against the highly virulent wild type, TAT. Immunogenicity tests carried out on fish vaccinated with inactivated vaccines were however not different in terms of protection attained for the TAT, TTT, and PTA inactivated vaccines. This finding confirms our previous report [35] that showed that oil-adjuvanted, inactivated TAT and PTA vaccines conferred equal level of protection against mortality when challenged with the highly virulent TAT strain. While antibody levels in our previous study [35] were generally low and there was no significant difference between the TAT and PTA vaccinated fish in antibody levels, the present study showed significant differences in VN titers and ELISA antibody levels between TAT and PTA vaccines. It has been shown that adjuvants can beneficially alter the qualitative and quantitative nature of the adaptive immune response resulting in enhanced protection [36]. Hence, it is likely that a combined output of the specific (the antigen) and nonspecific moieties (the adjuvant) [37] of our inactivated vaccines enhanced protection for all groups thereby reducing the differences of PCSPs between vaccines. In contrast, immunogenicity tests for the live vaccines were predictive of PCSPs as shown that antibody levels of fish vaccinated with the PTA vaccine were five times lower than fish vaccinated with the TAT vaccine when tested against the TAT antigen at challenge (Fig. 5A). This corresponded to the ability to prevent post challenge tissue damage for the TAT (0.0%, *n* = 10) and PTA (40%, *n* = 10) live vaccines (Table S6). Further the risk of fish dying in the group vaccinated with the PTA vaccine was five times higher than for fish vaccinated with the TAT vaccine. These data show that in the absence of adjuvants, PCSPs are higher for the corresponding antigen to the challenge virus. This befits the common observation made for viruses prone to high antigenic mutations. For influenza viruses, protection is often correlated with immunogenicity and it is now generally accepted that the antibody response measured against the strain causing the disease in an area is the surrogate marker for clinical efficacy in that area [38], [39]. From this and based on our findings, a logical consequence would be that vaccines against IPN in salmon in Norway should elicit immune responses against the virulent variant since this is the most frequently occurring genotype [22].

An interesting observation made from the live vaccine efficacy trial was that non-vaccinated control fish put to cohabit with TAT and PTA vaccinated fish showed differences in PCSPs that corresponded with protection levels observed in vaccinated fish. Data in Table S4 shows that PTA-controls cohabiting with the PTA vaccinated fish were at significantly higher risk of dying than TAT-controls cohabiting with TAT vaccinated fish. This corresponded with infection ratios of TAT-controls (40%, *n* = 10) compared to the PTA-controls (60%, *n* = 10) (Table S6). Although we did not monitor the concentration of virus in the tanks, a foreseeable explanation to this observation is that neutralizing antibodies in vaccinated fish could have reduced the quantity of circulating virus in the tanks. We cannot draw a firm conclusion but indications are that levels of protection in vaccinated fish had an impact on non-vaccinated control fish (in the same tanks), likely by reducing the challenge pressure equivalent to herd immunity. Given that there are no similar reports from previous IPNV efficacy trials there is need for more studies to verify the consistency and validity of these observations.

Data from inactivated vaccines show that antibodies are correlates of protection (r = −0.9590, p<0.021). Similar inverse correlations between hemagglutination inhibition (HI) antibody titers with post exposure influenza virus infections have been reported [40]–[42]. In one study [39] during a natural influenza A epidemic it was shown that the probability of clinical infection correlated with pre-epidemic homologous HI antibody titers and yet in another study, Davies and Grilli [38] showed that protection from infection was correlated with presence of antibodies homologous to the outbreak strain. Similarly, fish vaccinated with the homologous strain to the challenge virus in this study, had lowest post challenge infection implying that recall of the antigenic signature imprinted at vaccination was cardinal to reducing post challenge viral infections.

Residues that play a key role in virulence and immunogenicity of IPNV are localized on the central region of the VP2 capsid as indicated from previous studies [19], [25], [43]. As mentioned by Coulibaly et al [43], these residues do not participate in contacts influencing the folding of the VP2 capsid or interaction between subunits that stabilize the virion, but they strategically point outwardly suggesting that their main influence is attachment on target cells. For now, we have shown that naturally occurring mutations on positions 217, 221 and 247 strategically located on the surface loops of the VP2-HVRs are not only important for virulence and cell culture adaptation as previously reported [19], [25], [43], but they also seem to play an important role in the immunogenicity of IPNV. The combined output of two efficacy trials including live and inactivated vaccines all pointing to immunogenicity of IPNV being localized on the surface loops of the VP2-HVRs. This can impact vaccine development strategies and we envision that technologies like reverse genetics will drive the next generation of vaccines into structure-based targeted antigens.

## Supporting Information

Figure S1 **Study design for the inactivated vaccines.** Study design I included a three parallel tank system to assess the efficacy of inactivated, water-in-oil adjuvanted vaccines made from the four viral strains listed in Table 1. Each vaccine was allocated a total of 186 fish. After vaccination, 62 fish were transferred to each of the three parallel tanks 1–3, giving a total of 248 vaccinated fish in each of the parallel tanks. Thereafter, 186 fish were injected with phosphate buffered saline and divided equally between 3 tanks resulting in 62 fish in each of the parallel tanks. The total number of fish per tank was 310. At 6 weeks post vaccination (wpv), 6 fish from each vaccine group and 6 controls were sampled from each of the parallel tanks. This was repeated at 10 wpv leaving a total of 200 vaccinees and 50 controls per tank. At challenge 30 virus shedders injected with 1×10^7^ TCID_50_/ml of the TAT strain were put to cohabit with vaccinated and control fish in each tank. For each vaccine group, survivors of the challenge were pooled together and monitored for an additional 7 weeks.
(TIF)

Figure S2 **Study design for the live vaccines.** A three parallel tanks system including the virulent TAT and avirulent PTA vaccine strains. A total of 138 fish were injected with either of the live vaccines per group or given phosphate buffered saline (PBS control). After immune induction period, 38 vaccinated fish were transferred to each of three parallel tanks. To avoid cross infection since the study involved the use of live vaccines, each vaccine group was assigned its own parallel tank system. Also the PBS injected fish were kept separate from the vaccinated fish to avoid exposure to vaccine virus prior to challenge. At each sampling time point (4 &8 weeks pre challenge and 8&17 weeks post challenge), 4 fish from each group in each parallel tank were sacrificed and sampled. Challenge was carried out by adding eight virus shedders injected with 10^7^TCID_50_/ml and 30 PBS controls each parallel tank.
(TIF)

Figure S3 **Virus neutralization for the inactivated vaccines at 10** **weeks post challenge.** Virus neutralization (VN) antibody titers for the inactivated vaccines at 10 weeks post challenge (wpc) shows that the TTT vaccinated fish are significantly lower than all other vaccine groups while the TAT is not significantly higher than the PAA, PTA and control fish.
(TIF)

Figure S4 **Virus neutralization test for the live vaccine groups.** Virus neutralization antibody titers against the TAT strain for the TAT- and PTA-live vaccinated fish increase at 4 and 8 wpv.
(TIF)

Table S1 Hazard risk ratios of for inactivated vaccines expressed relative to the TAT vaccine. The data express the relative risk of dying in the TTT, PAA and PTA vaccinated fish relative to the TAT-vaccinated fish.
(DOCX)

Table S2 Relative risk of post challenge IPNV infection in head kidney samples of fish vaccinated with inactivated vaccines.
(DOCX)

Table S3 Relative risk of post challenge IPNV infection in head kidney samples of fish vaccinated with inactivated vaccines (Low-challenge dose; 1×10^6^TCID_50_/ml).
(DOCX)

Table S4 Post challenge hazard risk ratios of for live vaccines expressed relative to the TAT vaccine. The data express the relative risk of dying in the PTA, TAT-controls and PTA-controls relative to the TAT-vaccinated fish.
(DOCX)

Table S5 Post challenge virus re-isolation from head kidney samples of fish vaccinated with live vaccines.
(DOCX)

Table S6 Post challenge detection of viral antigens by immunohistochemistry and histopathology of fish vaccinated with live vaccines.
(DOCX)
