# Supplementary figures and images for: Immunogenicity and Cross Protective Ability of the Central VP2 Amino Acids of Infectious Pancreatic Necrosis Virus in Atlantic Salmon (Salmo salar L.)
Source: PLoS One. 2013 Jan 21;8(1):e54263. doi: 10.1371/journal.pone.0054263 (PMC3549989; doi:10.1371/journal.pone.0054263)

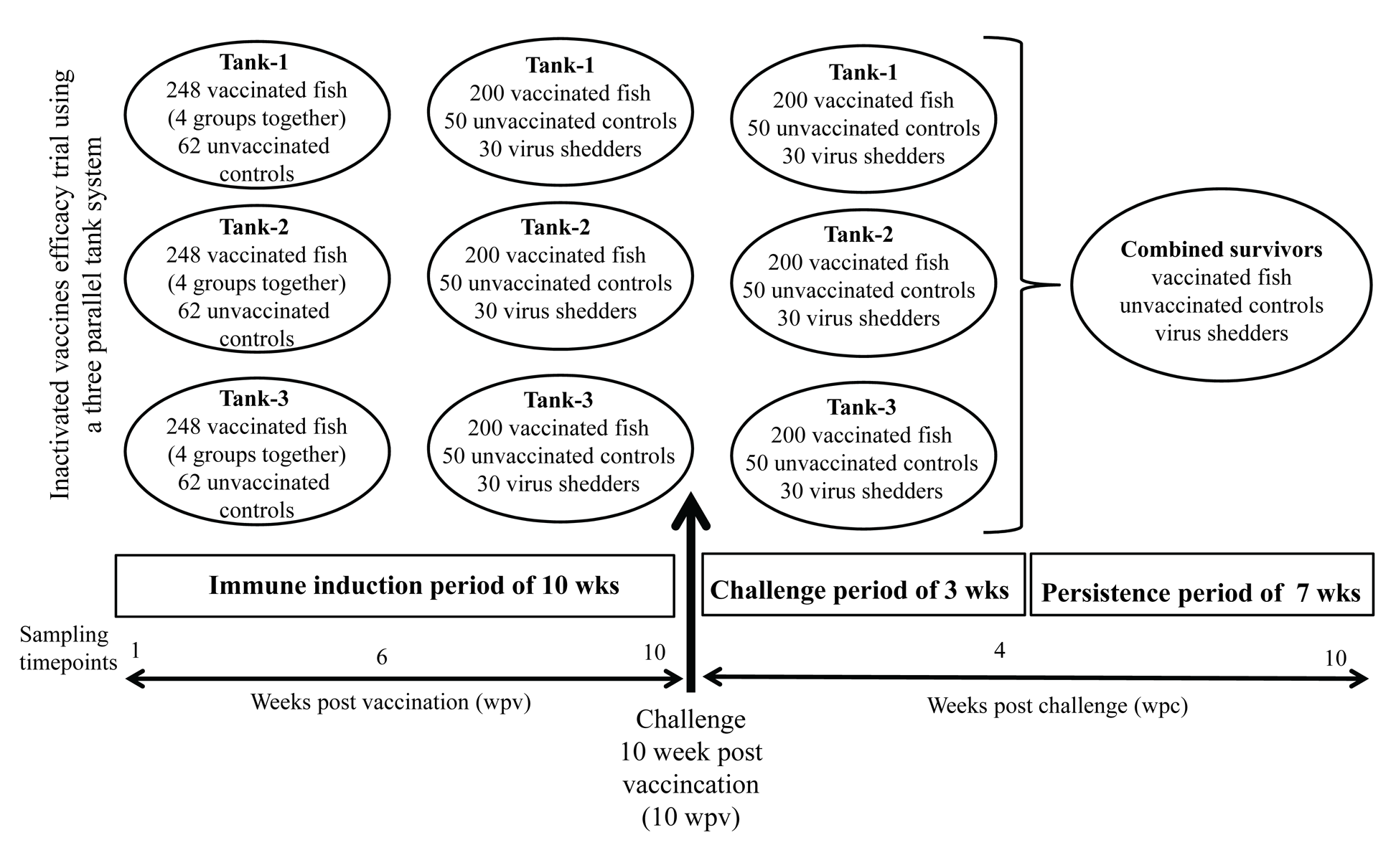

Supplement: Figure S1 — Study design for the inactivated vaccines. Study design I included a three parallel tank system to assess the efficacy of inactivated, water-in-oil adjuvanted vaccines made from the four viral strains listed in Table 1. Each vaccine was allocated a total of 186 fish. After vaccination, 62 fish were transferred to each of the three parallel tanks 1–3, giving a total of 248 vaccinated fish in each of the parallel tanks. Thereafter, 186 fish were injected with phosphate buffered saline and divided equally between 3 tanks resulting in 62 fish in each of the parallel tanks. The total number of fish per tank was 310. At 6 weeks post vaccination (wpv), 6 fish from each vaccine group and 6 controls were sampled from each of the parallel tanks. This was repeated at 10 wpv leaving a total of 200 vaccinees and 50 controls per tank. At challenge 30 virus shedders injected with 1×10^7^ TCID50/ml of the TAT strain were put to cohabit with vaccinated and control fish in each tank. For each vaccine group, survivors of the challenge were pooled together and monitored for an additional 7 weeks. (TIF) [file pone.0054263.s001.tif]

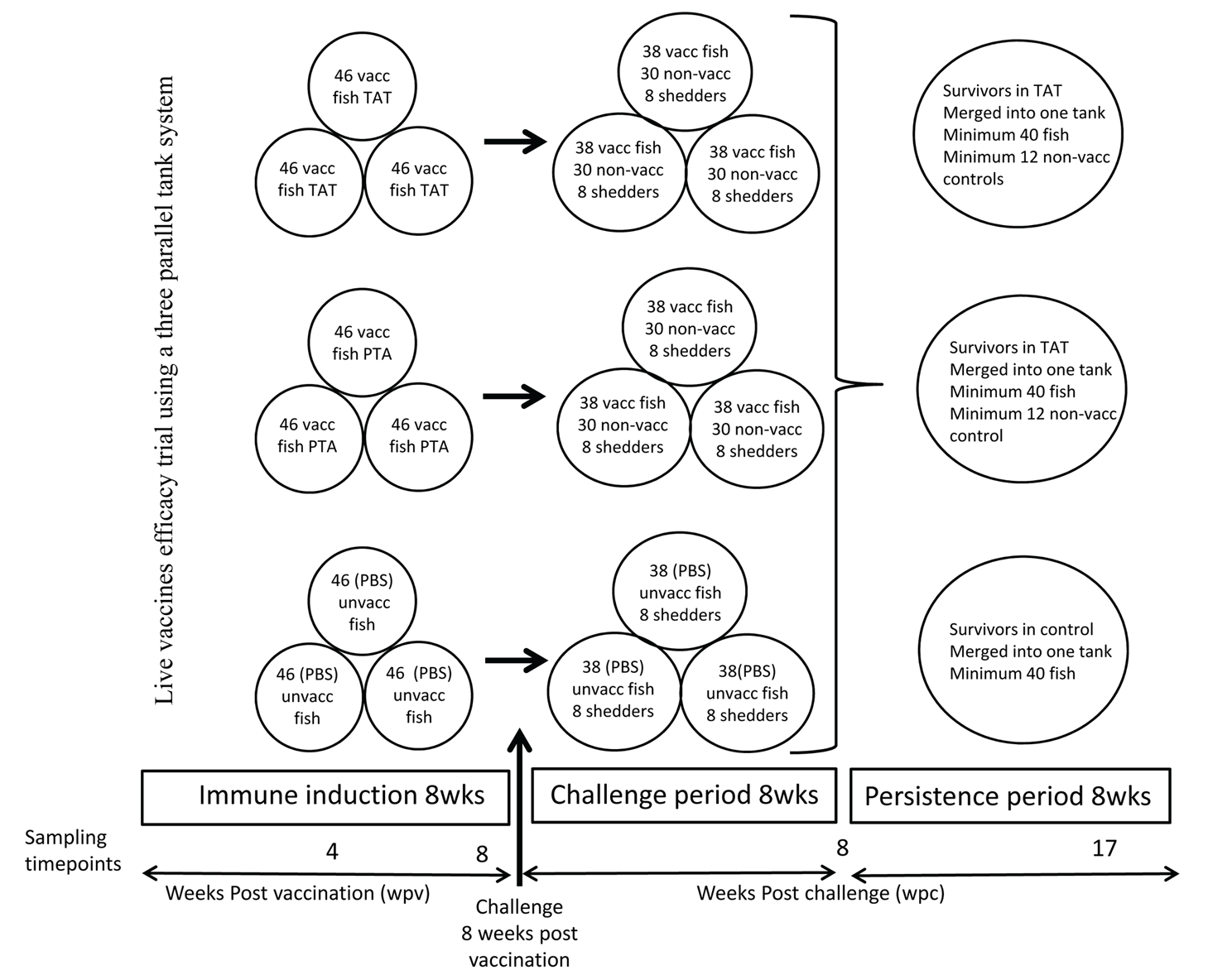

Supplement: Figure S2 — Study design for the live vaccines. A three parallel tanks system including the virulent TAT and avirulent PTA vaccine strains. A total of 138 fish were injected with either of the live vaccines per group or given phosphate buffered saline (PBS control). After immune induction period, 38 vaccinated fish were transferred to each of three parallel tanks. To avoid cross infection since the study involved the use of live vaccines, each vaccine group was assigned its own parallel tank system. Also the PBS injected fish were kept separate from the vaccinated fish to avoid exposure to vaccine virus prior to challenge. At each sampling time point (4 &8 weeks pre challenge and 8&17 weeks post challenge), 4 fish from each group in each parallel tank were sacrificed and sampled. Challenge was carried out by adding eight virus shedders injected with 107TCID50/ml and 30 PBS controls each parallel tank. (TIF) [file pone.0054263.s002.tif]

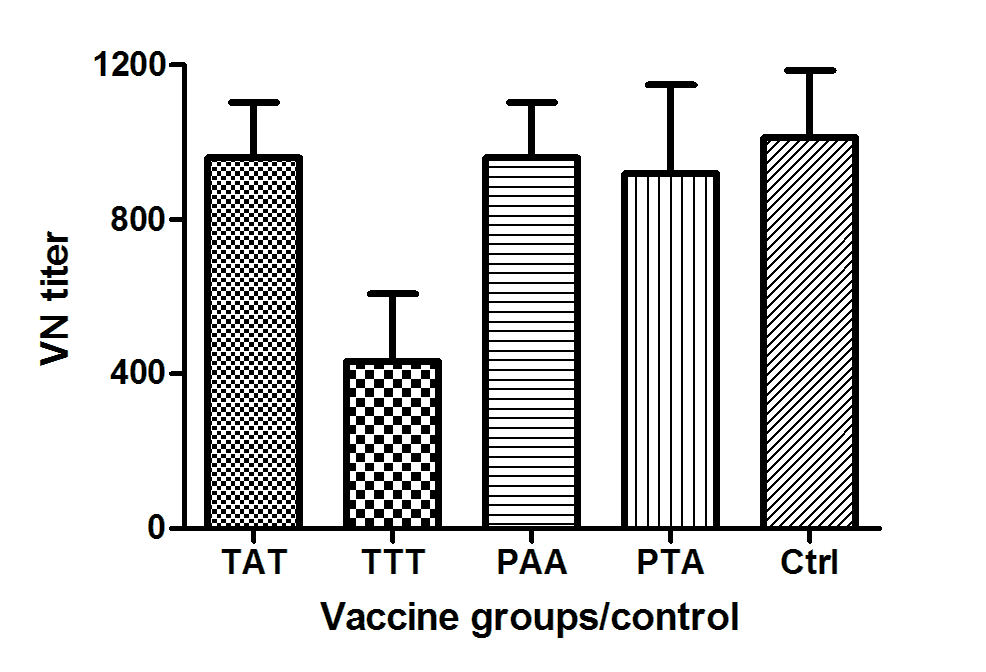

Supplement: Figure S3 — Virus neutralization for the inactivated vaccines at 10 weeks post challenge. Virus neutralization (VN) antibody titers for the inactivated vaccines at 10 weeks post challenge (wpc) shows that the TTT vaccinated fish are significantly lower than all other vaccine groups while the TAT is not significantly higher than the PAA, PTA and control fish. (TIF) [file pone.0054263.s003.tif]

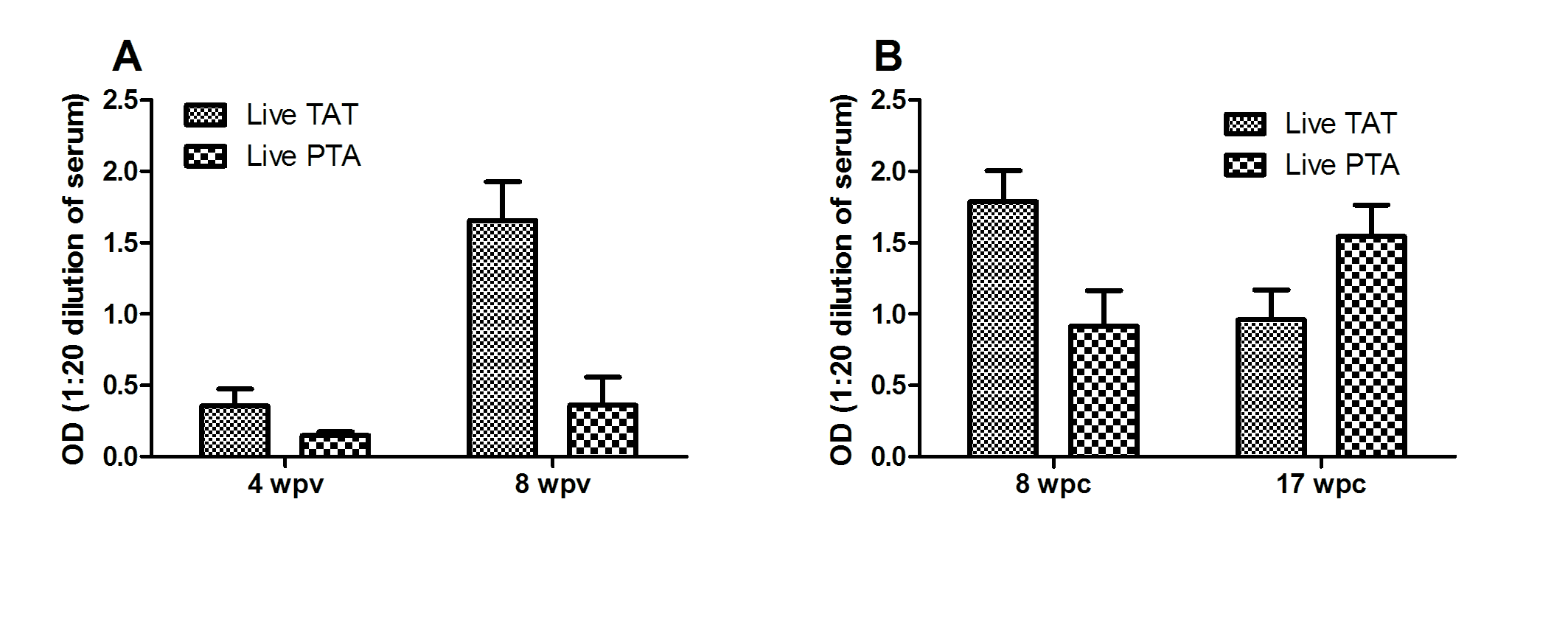

Supplement: Figure S4 — Virus neutralization test for the live vaccine groups. Virus neutralization antibody titers against the TAT strain for the TAT- and PTA-live vaccinated fish increase at 4 and 8 wpv. (TIF) [file pone.0054263.s004.tif]
