# Supplementary material for: Immunogenicity and Cross Protective Ability of the Central VP2 Amino Acids of Infectious Pancreatic Necrosis Virus in Atlantic Salmon (Salmo salar L.)
Source: PLoS One. 2013 Jan 21;8(1):e54263. doi: 10.1371/journal.pone.0054263 (PMC3549989; doi:10.1371/journal.pone.0054263)
Supplement: Table S1 — Hazard risk ratios of for inactivated vaccines expressed relative to the TAT vaccine. The data express the relative risk of dying in the TTT, PAA and PTA vaccinated fish relative to the TAT-vaccinated fish. (DOCX) [file pone.0054263.s005.docx]

**Table S1**. Hazard risk ratios of for inactivated vaccines expressed relative to the TAT vaccine. The data express the relative risk of dying in the TTT, PAA and PTA vaccinated fish relative to the TAT-vaccinated fish.

| **Vaccine Groups** | **HR** | **St error** | **Z** | **P-value** | **95% Conf intervals** |
| --- | --- | --- | --- | --- | --- |
| TTT | 0.9928 | 0.5307 | -0.01 | 0.989 | 0.3482 - 2.8304 |
| PAA | 2.4906 | 1.1186 | 2.03 | 0.042 | 1.0328 - 6.0063 |
| PTA | 1.4574 | 0.7182 | 0.76 | 0.445 | 0.5547 - 3.8829 |
| Control | 10.1213 | 4.1458 | 5.65 | 0.000 | 4.5350 - 22.5890 |

All hazard risk ratios are expressed relative to the TAT vaccinated group (RPS=88.33%). HR-hazard risk; St error – standard error; conf intervals – confidence intervals.
