# Supplementary material for: Immunogenicity and Cross Protective Ability of the Central VP2 Amino Acids of Infectious Pancreatic Necrosis Virus in Atlantic Salmon (Salmo salar L.)
Source: PLoS One. 2013 Jan 21;8(1):e54263. doi: 10.1371/journal.pone.0054263 (PMC3549989; doi:10.1371/journal.pone.0054263)
Supplement: Table S2 — Relative risk of post challenge IPNV infection in head kidney samples of fish vaccinated with inactivated vaccines. (DOCX) [file pone.0054263.s006.docx]

**Table S2**. Relative risk of post challenge IPNV infection in headkidney samples of fish vaccinated with inactivated vaccines.

| **Time Point** | **Vaccine strain** | **Fish examined (*n*)** | **Infected fish** | **Relative Risk** | **95% Conf. Interval** | **P-value** |
| --- | --- | --- | --- | --- | --- | --- |
| 4 wpc | TAT | 12 | 3 | 0.2609 | 0.0977 – 0.6963 | 0.0073 |
|  | TTT | 12 | 10 | 1.0256 | 0.7696 – 1.3669 | 0.8696 |
|  | PAA | 12 | 12 | 1.2564 | 1.0396 – 1.5184 | 0.0182 |
|  | PTA | 12 | 12 | 1.2564 | 1.0396 – 1.5184 | 0.0182 |
|  | Control | 12 | 12 | 1.2564 | 1.0396 – 1.5184 | 0.0182 |
| 10 wpc | TAT | 12 | 5 | 0.5128 | 0.2590 – 1.0154 | 0.0092 |
|  | TTT | 12 | 8 | 0.8889 | 0.5770 – 1-3694 | 0.5932 |
|  | PAA | 12 | 9 | 1.0285 | 0.7109 – 1.4882 | 0.8812 |
|  | PTA | 12 | 10 | 1.3333 | 1.0323 – 1.7222 | 0.0276 |
|  | Control | 12 | 12 | 1.4497 | 1.1551 – 1.8195 | 0.0014 |

WPC= weeks post challenge.
