# Supplementary material for: Immunogenicity and Cross Protective Ability of the Central VP2 Amino Acids of Infectious Pancreatic Necrosis Virus in Atlantic Salmon (Salmo salar L.)
Source: PLoS One. 2013 Jan 21;8(1):e54263. doi: 10.1371/journal.pone.0054263 (PMC3549989; doi:10.1371/journal.pone.0054263)
Supplement: Table S3 — Relative risk of post challenge IPNV infection in head kidney samples of fish vaccinated with inactivated vaccines (Low-challenge dose; 1×10^6^TCID50/ml). (DOCX) [file pone.0054263.s007.docx]

**Table S3**. Relative risk of post challenge IPNV infection in headkidney samples of fish vaccinated with inactivated vaccines (Low-challenge dose; 1 x10^6^TCID_50_/ml)

| **Time Point** | **Vaccine strain** | **Fish examined (*n*)** | **Infected fish** | **Relative Risk** | **95% Conf. Interval** | **P-value** |
| --- | --- | --- | --- | --- | --- | --- |
| 6 wpc | TAT | 12 | 0 | 0.0802 | 0.0052 – 1.2341 | 0.0704 |
|  | TTT | 12 | 3 | 0.6000 | 0.2130 – 1.6901 | 0.3336 |
|  | PAA | 12 | 5 | 1.1111 | 0.5183 – 2.3821 | 0.7866 |
|  | PTA | 12 | 5 | 1.1111 | 0.5183 – 2.3821 | 0.7866 |
|  | Control | 12 | 10 | 3.0769 | 1.8135 – 5.2206 | 0.0000 |
| 10 wpc | TAT | 12 | 3 | 0.2727 | 0.1020 – 0.7294 | 0.0096 |
|  | TTT | 12 | 10 | 1.0811 | 0.8038 – 1.4540 | 0.6061 |
|  | PAA | 12 | 11 | 1.2222 | 0.9651 – 1.5478 | 0.0958 |
|  | PTA | 12 | 11 | 1.2222 | 0.9651 – 1.5478 | 0.0958 |
|  | Control | 12 | 12 | 1.3277 | 1.0822 – 1.6276 | 0.0065 |

Only one tank was used for this study. WPC = weeks post challenge.
