# Supplementary material for: Immunogenicity and Cross Protective Ability of the Central VP2 Amino Acids of Infectious Pancreatic Necrosis Virus in Atlantic Salmon (Salmo salar L.)
Source: PLoS One. 2013 Jan 21;8(1):e54263. doi: 10.1371/journal.pone.0054263 (PMC3549989; doi:10.1371/journal.pone.0054263)
Supplement: Table S4 — Post challenge hazard risk ratios of for live vaccines expressed relative to the TAT vaccine. The data express the relative risk of dying in the PTA, TAT-controls and PTA-controls relative to the TAT-vaccinated fish. (DOCX) [file pone.0054263.s008.docx]

**Table S4**. Post challenge hazard risk ratios of for live vaccines expressed relative to the TAT vaccine. The data express the relative risk of dying in the PTA, TAT-controls and PTA-controls relative to the TAT-vaccinated fish.

| **Vaccine Group** | **HR** | **St error** | **Z** | **P-value** | **95%Conf. Interval** |
| --- | --- | --- | --- | --- | --- |
| PTA-live | 4.7454 | 1.9923 | 3.71 | 0.000 | 2.0841-10.8052 |
| TAT-live-Co | 8.4280 | 3.3204 | 5.14 | 0.000 | 3.6616-18.1079 |
| PTA-live-Co | 13.8165 | 5.4975 | 6.60 | 0.000 | 6.3344-30.1361 |
| Control | 29.3723 | 11.6769 | 8.50 | 0.000 | 13.4755-64.0222 |

All hazard risk ratios are expressed relative to the TAT vaccinated group (RPS=92.22%). HR – hazard risk; St error – standard error; conf. interval – confidence interval. TAT-live-Co and PTA-live-Co are the non-vaccinated control fish that cohabited with the respective vaccine groups post challenge.
