# Supplementary material for: Immunogenicity and Cross Protective Ability of the Central VP2 Amino Acids of Infectious Pancreatic Necrosis Virus in Atlantic Salmon (Salmo salar L.)
Source: PLoS One. 2013 Jan 21;8(1):e54263. doi: 10.1371/journal.pone.0054263 (PMC3549989; doi:10.1371/journal.pone.0054263)
Supplement: Table S5 — Post challenge virus re-isolation from head kidney samples of fish vaccinated with live vaccines. (DOCX) [file pone.0054263.s009.docx]

**Table S5**. Post challenge virus re-isolation from headkidney samples of fish vaccinated with live vaccines

| **Vaccine code** | **Total Per Group (*n*)** | **Days Post vaccination** | | |  | **Days Post challenge** | |
| --- | --- | --- | --- | --- | --- | --- | --- |
|  |  | **Day 0** | **4 wpv** | **8 wpv** |  | **8 wpc** | **17 wpc** |
| TAT-Live | 12 | 0 | 12 | 12 |  | 10 | 8 |
| PTA-Live | 12 | 0 | 12 | 12 |  | 10 | 8 |
| TAT-live-Co | 12 | 0 | 0 | 0 |  | 11 | 3/3 |
| PTA-Live-Co | 12 | 0 | 0 | 0 |  | 11 | 3/3 |
| Control | 12 | 0 | 0 | 0 |  | 11 |  |

*n*=total number of fish examined per group. TAT-live-Co and PTA-live-Co are the non-vaccinated control fish that cohabited with the respective vaccine groups post challenge. wpv-weeks post vaccination; wpc-weeks post challenge.
