# Supplementary material for: Immunogenicity and Cross Protective Ability of the Central VP2 Amino Acids of Infectious Pancreatic Necrosis Virus in Atlantic Salmon (Salmo salar L.)
Source: PLoS One. 2013 Jan 21;8(1):e54263. doi: 10.1371/journal.pone.0054263 (PMC3549989; doi:10.1371/journal.pone.0054263)
Supplement: Table S6 — Post challenge detection of viral antigens by immunohistochemistry and histopathology of fish vaccinated with live vaccines. (DOCX) [file pone.0054263.s010.docx]

**Table S6**. Post challenge detection of viral antigens by immunohistochemistry and histopathology of fish vaccinated with live vaccines

|  | 8 Weeks Post Vaccination | | | | | | |  | | 8 Weeks Post Challenge | | | | | |
| --- | --- | --- | --- | --- | --- | --- | --- | --- | --- | --- | --- | --- | --- | --- | --- |
|  | *n* | H-kidney | Spleen | Liver | Pancreas | Overall infection (%) |  | | *n* | | H-kidney | Spleen | Liver | Pancreas | Overall infection (%) |
| TAT-live | 6 | 0 | 0 | 0 | 0 | 0 |  | | 10 | | 0 | 0 | 0 | 0 | 0 (0.0%) |
| PTA-live | 6 | 0 | 0 | 0 | 0 | 0 |  | | 10 | | 1 | 1 | 1(1) | 3(3) | 4 (40.0) |
| Control | 6 | 0 | 0 | 0 | 0 | 0 |  | | 10 | | 5 | 5 | 6(6) | 7(7) | 7 (70.0%) |
| TAT-live-Co | - | - | - | - | - | - |  | | 10 | | 1 | 1 | 2(2) | 4(4) | 4(40.0%) |
| PTA-live-Co | - | - | - | - | - | - |  | | 10 | | 3 | 3 | 4(4) | 6(6) | 6 (60.0%) |

*n*= total number of fish examined per group. H-kidney= Headkidney. TAT-live-Co and PTA-live-Co are the non-vaccinated control fish that cohabited with the respective vaccine groups post challenge
